# Petrography and Physicomechanical Properties of Rocks from the Ambela Granitic Complex, NW Pakistan

**DOI:** 10.1155/2013/349381

**Published:** 2013-06-02

**Authors:** Mohammad Arif, S. Wajid Hanif Bukhari, Noor Muhammad, Muhammad Sajid

**Affiliations:** ^1^Department of Geology, University of Peshawar, Peshawar 25120, Pakistan; ^2^Centre of Excellence in Mineralogy, University of Balochistan, Quetta 87300, Pakistan; ^3^Department of Mining Engineering, NWFP University of Engineering and Technology, Peshawar 25120, Pakistan

## Abstract

Petrography and physicomechanical properties of alkali granites, alkali quartz syenite, and nepheline syenite from Ambela, NW Pakistan, have been investigated. Whereas the alkali quartz syenite and most of the alkali granites are megaporphyritic, the nepheline syenite and some of the alkali granites are microporphyritic. Their phenocryst shape and size and abundance of groundmass are also different. The values of unconfined compressive strength (UCS) are the lowest and highest for megaporphyritic alkali granite and alkali quartz syenite, respectively. However, all the four rock types are moderately strong. Correspondingly, their specific gravity and water absorption values are within the permissible range for use as construction material. The UCS for the alkali quartz syenite is the highest, most probably because (i) it has roughly equal amounts of phenocryst and groundmass, (ii) it displays maximum size contrast between phenocryst and groundmass, (iii) its phenocrysts are highly irregular, and (iv) it contains substantial amounts of quartz.

## 1. Introduction

**Figure 1:**
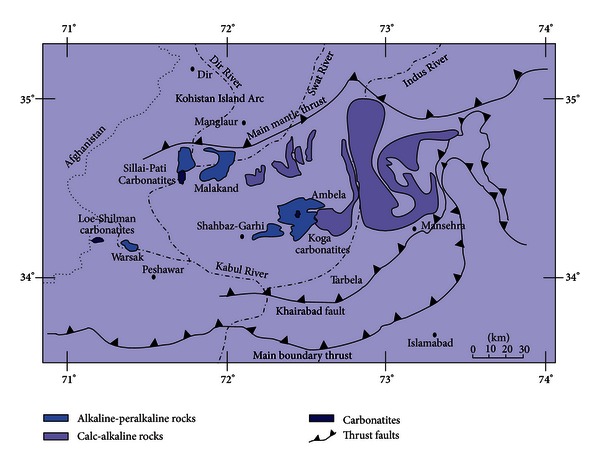
Generalized geological map showing the location of alkaline rocks in northern Pakistan; redrawn from Khattak et al. [22].

The middle to late Paleozoic Ambela Granitic Complex (AGC) is a part of the Peshawar plain alkaline igneous province (PPAIP) [1–3] and lies in NW Pakistan (Figure 1). The rocks of this composite batholith have been distinguished into several types [4]. The focus of the present study is to give an account of petrographic characteristics and physicomechanical properties of representative samples of alkali granite, quartz syenite, and nepheline syenite from the AGC. An attempt has also been made to correlate mechanical properties of the mentioned rocks with their petrographic details. It is important to mention that a number of previous workers, for example Din and Rafiq [5] have also determined the mechanical properties of rocks from different places in NW Pakistan including Ambela.

## 2. Geology and Tectonics

The northern part of Pakistan consists of (i) the Eurasian plate in the north, (ii) Kohistan island arc in the middle, and (iii) Indo-Pakistan plate in the south [6, 7]. The PPAIP constitutes a major magmatic activity zone within the northwestern margin of the Indo-Pakistan plate. It stretches over a distance of 150 to 200 km along the northern and northwestern margins of the Peshawar plain, from the Pak-Afghan border in the west to Tarbela in the east (Figure 1). Several types of alkaline igneous rocks including the ones from the AGC constitute the PPAIP.

The AGC consists of silica oversaturated (70%), silica saturated (20%), and silica undersaturated rocks (5%) and dykes (5%). These are classified into three groups on the basis of modal mineralogy [4]. Group 1 includes granites, alkali granites, and microporphyrites. Having been dated at 315 ± 15 Ma [3], they represent the earliest magmatic episode of the complex. Group 2 comprises quartz syenites, alkali quartz syenites, syenites (315 ± 15 Ma), feldspathoid syenite, and ijolite (297 ± 4 Ma; [3]), carbonatite, lamprophyre, and associated pegmatite and fenites. Group 3 consists of thin to fairly thick, black to green color dykes of basic to intermediate composition, that is, dolerite, metadolerite, and diorite cutting both the group 1 and group 2 rocks and thus representing the latest magmatic episode in the Ambela complex.

## 3. Methodology

Fresh (unaltered) bulk and fist-sized rock samples were collected. Two of the four bulk samples collected for determining physicomechanical properties represent alkali granites from Ambela proper, while the remaining two include one each of quartz syenite from Babaji Kandao and nepheline syenite from Koga area (Figure 1). Thin sections from all the bulk and fist-size samples were prepared for petrographic studies in the Department of Geology, University of Peshawar, Pakistan.

For determining mechanical properties, two cylindrical core samples were obtained from each of the bulk samples with the help of a core drilling machine in the Rock Mechanics laboratory of the Department of Mining Engineering, NWFP University of Engineering and Technology, Peshawar, Pakistan. All the core samples were subjected to (i) unconfined compressive strength (UCS), (ii) unconfined tensile strength (UTS), and (iii) shear strength. Besides, specific gravity and capacity of water absorption of all the bulk samples were determined in the Geochemistry Laboratory of the National Center of Excellence in Geology, University of Peshawar, Pakistan.

## 4. Petrography

Fifteen representative rock thin sections were used for a detailed petrographic investigation including structural/textural characteristics, identification of the constituent mineral phases, and determination of modal mineralogical composition through visual estimation. Based on these details, the studied rocks are distinguished into the following types.

### 4.1. Alkali Granite

Investigation of nine representative thin sections leads to a further subdivision of the alkali granite into two textural types: (i) megaporphyritic and (ii) microporphyritic.

#### 4.1.1. Megaporphyritic Alkali Granites

**Figure 2:**
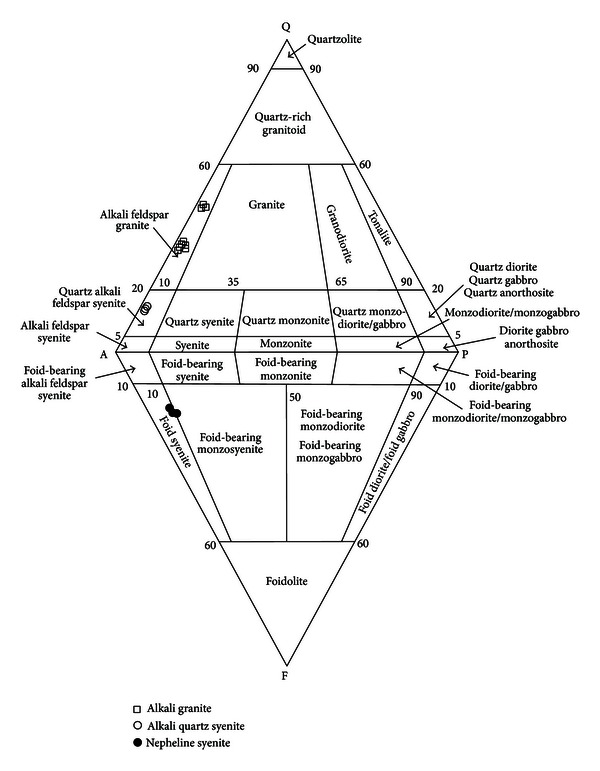
Modal composition of the studied rocks from the Ambela complex plotted on the Q (quartz)-A (alkali feldspar)-P (plagioclase)-F (foid) diagram of Streckeisen [23].

On the basis of the relative proportions (volume %) of the three essential minerals, that is, perthitic alkali feldspar (50–55%), quartz (28–33%), and plagioclase (2-3%), these largely hypidiomorphic and inequigranular to subequigranular rocks fall within the compositional field of alkali feldspar granite (Figure 2). A variety of accessory minerals, including biotite, muscovite, sphene, zircon, garnet, and epidote, also occur in these rocks. However, the collective modal abundance of all these does not exceed 15%. Among these, biotite is the most common and abundant (trace amounts to 8%) that usually occurs in clusters with muscovite (ranging up to 2 modal %), chlorite, sphene, and opaque ore mineral.

**Figure 3:**
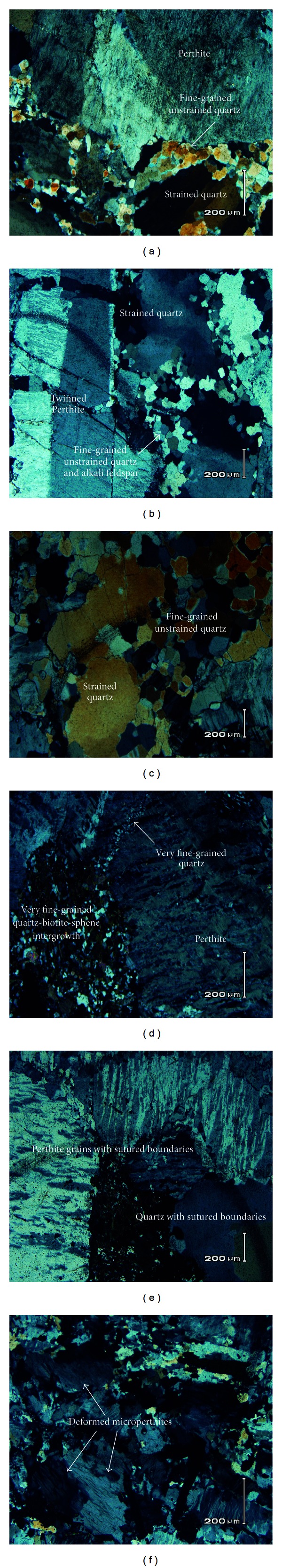
Photomicrographs showing important petrographic features in the investigated samples of megaporphyritic alkali granite (a and b), microporphyritic alkali granite (c), megaporphyritic alkali quartz syenite (d and e) and nepheline syenite (f). See text and Table 3 for details.

The alkali feldspar, including microcline, is mostly perthitic and occurs as megacrysts that occasionally display simple twinning (Figures 3(a) and 3(b)). Microfractures in the perthite grains contain fine-grained alkali feldspar. At places, the alkali feldspar grains show partial alteration to clay mineral(s). Quartz occurs as small-to-medium-sized subhedral to anhedral grains. The relatively larger grains of quartz show patchy or wavy extinction, while the fine-grained quartz displays uniform extinction (Figure 3(a)).

#### 4.1.2. Microporphyritic Alkali Granite

This inequigranular (to subequigranular) rock is hypidiomorphic to allotriomorphic. Perthitic alkali feldspar (41-42%), quartz (40–42%), and plagioclase (2-3%) are their essential constituents while biotite, garnet, muscovite, apatite, sphene, and zircon are the commonly occurring accessory minerals. In addition to being finer grained, the rock under discussion shows significantly higher quartz to feldspar ratio than the one described above (Figure 2). Most of its other petrographic features, especially the optical properties, grain size distribution and mode of occurrence of quartz (Figure 3(c)), and type and relative abundance of the mafic minerals, are similar to those of the megaporphyritic variety.

### 4.2. Megaporphyritic Alkali Quartz Syenite

Investigation of three thin sections suggests that this rock predominantly consists of perthitic alkali feldspar (64–66 modal %), which occurs in two distinct grain-size populations, namely, coarse-grained and medium to fine grained. The latter together with microcline, quartz (8–10%) and plagioclase (1-2%) constitutes the matrix to the megacrysts/phenocrysts that almost totally consist of perthite. Amphibole, biotite, opaque ore mineral, sphene, epidote, and aegirine augite are the commonly occurring mafic minerals, which collectively constitute about 25% by volume of the rock. Of these, amphibole is one of the major constituents and mostly the second most abundant mineral (averaging ~12%). The abundance of sphene is also substantial (4-5%) while all the other mafic minerals and carbonate (1-2%) occur in minor-to-accessory amounts. Apatite, zircon, monazite, and fluorite occur in traces.

The total abundance of mafic minerals and feldspar to quartz ratio in the porphyritic alkali quartz syenite is distinctly higher but micas much less abundant than in alkali granites. The alkali quartz syenite shows close similarity to the megaporphyritic alkali granite in terms of the form and size of grains and other textural features of perthitic alkali feldspar and plagioclase. However, perthite is largely unaltered in the former type.

Quartz largely occurs as fine- to very fine-grained groundmass and along the boundaries of perthite. Aggregates of very fine-grained quartz occur in association with biotite, anhedral sphene, and amphibole (Figures 3(d) and 3(e)). Fine globules and worm-like patches of quartz also occur in association with and within perthite grains forming myrmekite or granophyric texture.

### 4.3. Nepheline Syenite

Study of three representative thin sections reveals that the nepheline syenite essentially consists of alkali feldspar (54–57%), nepheline (14–16%), hornblendic amphibole (6–9%), sphene (5-6%), plagioclase (4-5%) and aegirine augite (4-5%). The commonly occurring accessory minerals include biotite, opaque ore mineral, apatite, zircon, and monazite. All the nonfelsic minerals collectively constitute about 25% by volume of the studied samples. Both the amphibole and pyroxene show alteration into chlorite and/or epidote.

The alkali feldspar (and to a less extent microcline) is largely medium grained and occurs as tabular and mostly subhedral perthitic grains showing the effects of kaolinization. Some of the perthitic alkali-feldspar grains show simple twinning. Nepheline is medium to fine grained and euhedral to subhedral in form. It also occurs in crystals with prismatic habit. The margins, shape, and disposition of nepheline grains as well as those of perthite and plagioclase suggest that the rock has been deformed (Figure 3(f)).

## 5. Mechanical and Physical Properties

### 5.1. Strength

The UCS and UTS of the petrographically investigated samples of alkali granites, alkali quartz syenite, and nepheline syenite were determined in the laboratory. Besides, the values of shear strength were also worked out. UCS and UTS were measured directly by strength testing machine while cohesion and angle of internal friction, which collectively determine shear strength, were derived from the values of UCS and UTS. Relevant information including definitions of these various tests and details regarding the nature and preparation of samples and different methods used for their determination and calculation has been outlined elsewhere [8].

Two core samples per bulk sample were used for determining the UCS and UTS. The values of UCS of all the studied rocks, including even the megaporphyritic alkali granite whose UCS value is the lowest, are high enough (27–47 MPa; Table 1) to group them with the moderately strong category of Anon [9–11]. It is generally believed that UCS of rocks is 8–10 times their UTS [12, 13]. The UCS to UTS ratios of almost all the studied samples fall within this range.

### 5.2. Water Absorption

Determination of water absorption is important as repeated hydration and dehydration result in mechanical disruption of small portions of rock close to an exposed surface and allow access of water into the rock and thus causing an increase in the degree and rate of weathering [14]. Employing the method and calculations described elsewhere [8], the values of water absorption for the studied samples of alkali granites, alkali quartz syenite, and nepheline syenite were determined (Table 2). As expected, the UCS and water absorption values display a somewhat negative correlation.

### 5.3. Specific Gravity

It has been found that the rate of softening of rock specimens on immersion in water depends on their origin [15]. However, they swell slowly hence decreasing density and strength. The resulting loss in strength is very significant in controlling the engineering properties of rocks. The rocks having specific gravity ≥2.55 are considered to be suitable for heavy construction work [16].

The specific gravity of the mega-porphyritic alkali granite, micro-porphyritic alkali granite, alkali quartz syenite and nepheline syenite samples was determined in the laboratory using the equipment and formula mentioned elsewhere [8]. The values obtained are given in Table 2.

## 6. Discussion and Conclusions

A detailed petrographic investigation reveals that the studied samples from the Ambela complex represent three mineralogically different rock types, namely, alkali granite, alkali quartz syenite, and nepheline syenite (Figure 2). In addition to differences in modal mineralogy, these rocks also differ markedly in terms of their textural details, especially grain size and shapes.

All the rocks are more or less inequigranular and hence termed porphyritic. However, there are significant differences among them with respect to size and shape of the contained phenocrysts as well as relative proportion and grain size of the groundmass (Table 3). Whereas the phenocrysts in nepheline syenite are fine to medium grained, those in the samples of alkali quartz syenite and most of the alkali granite are very coarse grained. Furthermore, phenocrysts in three of the studied alkali granite samples are medium to fine grained.

The investigated rocks also differ significantly in terms of type and abundance of the nonfelsic constituents. The total amount of mafic phases in both the alkali quartz syenite and nepheline syenite (~25 modal %) is distinctly higher than the alkali granite (~15% mafic minerals). Whereas the most abundant mafic phases in the alkali granites are biotite and opaque ore mineral, amphibole and sphene constitute the same in both the alkali quartz syenite and nepheline syenite. It should be noted, however, that mica flakes in the studied samples do not display any alignment and mostly occur in patches together with the other mafic minerals.

Although significantly different from one another, the UCS values of all the studied rocks fall within the range of those designated as moderately strong (Table 1). Similarly, the values of their water absorption are low and specific gravity high enough to render them suitable for use as construction material. More importantly, their values of water absorption and specific gravity are strongly related to each other and to the corresponding average values of UCS (Table 2).

The generally agreed upon geotechnically important features of rocks include (i) modal mineralogical composition, (ii) grain size and extent of its variation, (iii) grain shape, (iv) the overall abundance of flaky minerals (mica) and, more importantly, the degree of their preferred orientation, and (v) degree of weathering or alteration [8, 12, 14, 17–21]. Rocks containing large amounts of physically competent minerals are obviously strong. Similarly, rocks with finer grain size are stronger than those with similar modal mineralogy but coarser grain size. A wide range of within-rock grain size variation is also supposed to add to the strength of rock. Furthermore, rocks whose constituent mineral grains are irregularly shaped are likely to be stronger than otherwise similar ones but composed of grains with regular shape. 

As all the investigated rocks represent the same area and igneous activity of broadly similar age, they would hardly, if at all, differ in terms of their degree of weathering/alteration. The thin section observation also precludes any significant weathering or alteration of the studied samples. The general scarcity and more importantly the random orientation and patchy distribution of micas in the studied rocks eliminate any possible adverse effect on their strength and mechanical properties due to the fourth of the factors listed above. In other words, the difference in the values of UCS, specific gravity, and water absorption of the investigated samples can only be ascribed to differences in their modal mineralogy and texture.

The UCS values of the investigated rocks appear to be depending more on grain size than modal mineralogy. That is why the samples of alkali granite with broadly similar mineralogical composition but different phenocryst size have different values of UCS. The alkali granite with medium- to fine-grained phenocrysts is stronger (UCS = 35.849–39.622 MPa) than the alkali granite with very coarse-grained phenocrysts (UCS = 27.672–30.817 MPa). However, the UCS of alkali quartz syenite is the highest (47.169-45.911 MPa) although its average phenocrysts' size is comparable to that of the very coarse-grained alkali granite. Similarly, although the average size of phenocrysts in the nepheline syenite sample is the smallest (fine to medium), yet its UCS is low (30.188–34.591 MPa) and higher only than the alkali granite containing very large phenocrysts.

The difference between the average size of phenocrysts and that of the groundmass in individual samples increases from the mega-porphyritic alkali granite through nepheline syenite to quartz syenite. This is also the order of increase in the UCS values. Again, however, there is an exception; that is, the sample of alkali granite with medium- to fine-grained phenocrysts does not follow the otherwise positive relationship between the UCS and the grain size contrast between the phenocryst and the groundmass.

The ratio in the proportion of phenocrysts to matrix does not vary systematically with UCS. This ratio is the highest for the mega-porphyritic alkali granite and the lowest for the nepheline syenite. On the other hand, the UCS value of quartz syenite is the maximum, although the ratio between its phenocryst and matrix abundances is intermediate. Also, the alkali granite with medium- to small-sized phenocrysts and minimum phenocrysts to matrix ratio, like the mega-porphyritic alkali granite, has its UCS higher than both the latter and the nepheline syenite.

The irregularity displayed by the phenocryst shapes in quartz syenite is higher than that in both the mega-porphyritic and microporphyritic types of the alkali granite but comparable to that in nepheline syenite, which is fine to medium grained. Hence the strength of the studied rocks does not show any systematic variation with the degree of irregularity of their phenocrysts.

Hence it follows that none of the petrographic parameters independently controls the strength of the rocks investigated. Rather, a number of petrographic features appear to have collectively contributed to determine the actual rock strength. Thus the sample of quartz syenite shows the highest UCS most probably because (i) it contains roughly equal amounts of groundmass and phenocryst, (ii) the gain size of its groundmass is finest and thus it displays the maximum contrast in the grain size of its phenocryst and groundmass components, (iii) the shapes of its phenocrysts are highly irregular, and (iv) it contains substantial amounts of quartz. It is important to mention that although the sample of nepheline syenite is comparable to quartz syenite in terms of the first and third of these features, yet its UCS is lower than that of the latter probably because (i) it is totally devoid of quartz and (ii) it displays less contrast in the grain size of its phenocryst and groundmass components (Table 3).

## Figures and Tables

**Table 1:** UCS, UTS, and shear strength of the studied samples.

S. #	Rock type	UCS (MPa)	UTS (MPa)	UCS : UTS	Cohesion (MPa)	Angle of internal friction (Φ)
(1)	Megaporphyritic alkali granite	27.672 30.817	3.758 3.015	7.4 10.2	11.00	14.25°
(2)	Microporphyritic alkali granite	35.849 39.622	3.486 3.486	10.3 11.4	12.35	18.75°
(3)	Megaporphyritic alkali quartz syenite	47.169 45.911	4.536 4.532	10.4 10.1	15.60	19.50°
(4)	Nepheline syenite	30.188 34.591	3.949 3.949	7.6 8.8	12.00	12.50°

**Table 2:** Values of water absorption (%) and specific gravity and their relationship with UCS.

S. #	Rock type	Water absorption	Specific gravity	Average UCS (MPa)
(1)	Megaporphyritic alkali granite	0.73	2.53	29.273 ± 2.262
(2)	Microporphyritic alkali granite	0.93	2.46	37.736 ± 2.668
(3)	Megaporphyritic alkali quartz syenite	0.29	2.56	46.540 ± 0.890
(4)	Nepheline syenite	0.14	2.55	32.390 ± 3.113

**Table 3:** Comparison of petrographic characteristics of the studied rocks.

Rock type	Megaporphyritic alkali granite	Microporphyritic alkali granite	Megaporphyritic alkali quartz syenite	Nepheline syenite
Phenocryst grain size	Very coarse grained	Medium grained	Very coarse grained	Medium grained
Groundmass grain size	Fine	Fine	Very fine	Fine
Phenocryst modal abundance	90%	90%	60%	50%
Phenocryst shape	Regular to irregular	Regular to irregular	Highly irregular	Irregular
